# NMR metabolomics identifies over 60 biomarkers associated with Type II Diabetes impairment in *db/db* mice

**DOI:** 10.1007/s11306-019-1548-8

**Published:** 2019-06-10

**Authors:** Marina Mora-Ortiz, Patricia Nuñez Ramos, Alain Oregioni, Sandrine P. Claus

**Affiliations:** 1 Department of Food and Nutritional Sciences, The University of Reading, Whiteknights Campus, P.O. Box 226, Reading, RG6 6AP UK; 2 Department of Twin Research, Kings’ College London, St Thomas’ Hospital Campus, Westminster Bridge Road, London, SE1 7EW UK; 3 Facultad de Medicina, Universidad de Extremadura, Campus de Badajoz, C.P. 06006 Badajoz, Spain; 4 MRC Biomedical NMR Centre, The Francis Crick Institute, 1 Midland Road, London, NW1 1AT UK

**Keywords:** Type II Diabetes, Metabolome, Nuclear magnetic resonance (NMR) spectroscopy, *db/db* mouse

## Abstract

**Introduction:**

The rapid expansion of Type 2 Diabetes (T2D), that currently affects 90% of people suffering from diabetes, urges us to develop a better understanding of the metabolic processes involved in the disease process in order to develop better therapies. The most commonly used model for T2D research is the *db/db* (BKS.Cg-Dock7 < m > +/+ Lepr < db >/J) mouse model. Yet, a systematic ^1^H NMR based metabolomics characterisation of most tissues in this animal model has not been published. Here, we provide a systematic organ-specific metabolomics analysis of this widely employed model using NMR spectroscopy.

**Objectives:**

The aim of this study was to characterise the metabolic modulations associated with T2D in *db/db* mice in 18 relevant biological matrices.

**Methods:**

High-resolution ^1^H-NMR and 2D-NMR spectroscopy were applied to 18 biological matrices of 12 *db/db* mice (WT control n = 6, *db/db *= 6) aged 22 weeks, when diabetes is fully established.

**Results:**

61 metabolites associated with T2D were identified. Kidney, spleen, eye and plasma were the biological matrices carrying the largest metabolomics modulations observed in established T2D, based on the total number of metabolites that showed a statistical difference between the diabetic and control group in each tissue (16 in each case) and the strength of the O-PLS DA model for each tissue. Glucose and glutamate were the most commonly associated metabolites found significantly increased in nine biological matrices. Investigated sections where no increase of glucose was associated with T2D include all intestinal segments (i.e. duodenum, jejunum, ileum and colon). Microbial co-metabolites such as acetate and butyrate, used as carbon sources by the host, were identified in excess in the colonic tissues of diabetic individuals.

**Conclusions:**

The metabolic biomarkers identified using ^1^H NMR-based metabolomics will represent a useful resource to explore metabolic pathways involved in T2D in the *db/db* mouse model.

**Electronic supplementary material:**

The online version of this article (10.1007/s11306-019-1548-8) contains supplementary material, which is available to authorized users.

## Introduction

Type II Diabetes (T2D, also known as non-insulin-dependent, or adult onset diabetes) is a complex metabolic disorder characterised by insulin resistance and systemic hyperglycaemia (Tai et al. 2015). Common associated co-morbidities include kidney failure, nerve damage, blindness and cardiovascular diseases caused by poorly controlled hyperglycaemia (Amin et al. 2010; Anavekar et al. 2004; Trautner et al. 1997). The dramatic rise in diabetes has become a world leading cause of concern as it currently affects 422 million adults and results in circa 1.5 million deaths directly attributed to diabetes each year (http://www.who.int/diabetes/en/); T2D represents around 90% of the cases. T2D is also an increasing clinical issue among children and adolescents, who suffer more aggressive complications than adults or paediatric T1D, including hypertension, proteinuria, peripheral and autonomic neuropathy, renal disease and retinopathy (Krakoff et al. 2003; Eppens et al. 2006; Yokoyama et al. 1997; Group 2012).

Systematic metabolomics characterisation of various research models such as rodents, chickens, pigs, humans and horses have been published in the past (Claus et al. 2008; Le Roy et al. 2016; Martin et al. 2007; Merrifield et al. 2011; Ndagijimana et al. 2009; Holmes et al. 1997; Escalona et al. 2015; Mora-Ortiz et al. 2019), but to date, a comprehensive metabolic phenotyping of the leptin receptor defective (*db/db*) T2D mouse model: BKS.Cg-Dock7 < m > +/+ Lepr < db >/J is missing. Previous reports have characterised relevant biological matrices such as urine, plasma and kidneys, showing an increase in glucose levels and modulations in the tricarboxylic acid cycle (TCA cycle), branched-chain amino acids (BCAAs) levels, homocysteine-methionine metabolism and ketone and fatty acid metabolism at different stages of the disease. However, a systematic metabolomics characterisation of this animal model in a large number of biological matrices has never been published. Therefore, we herein provide a useful resource to progress in the understanding of organ-specific metabolic alterations in established T2D in the *db/db* mouse model (Saadat et al. 2012; Wei et al. 2015; Gipson et al. 2008; Connor et al. 2010; Kim et al. 2016; Salek et al. 2007; Wei et al. 2015).

Here, we characterised the metabolic profiles of 18 biological matrices relevant to T2D pathology in the widely-used mouse model BKS.Cg-Dock7 < m > +/+ Lepr < db >/J.

## Materials and methods

### Animal handling and sample collection

In order to characterise the metabolic fingerprint of T2D, twelve four-week-old mice (females, n = 8; males, n = 4) from the strain BKS.Cg-Dock7 < m > +/+ Lepr < db >/J and their corresponding WT controls were acquired from Charles River Laboratories, Italy. Animals were allocated into two different homogenous environments, diabetic and control, according to their genetic background (*db/db *= 6, of which 4 were females and 2 males; control = 6, of which 4 were females and two were males) and bedding from each environment was mixed on weekly basis to minimise cage effect. After one week of acclimatisation, body weight was recorded on a weekly basis starting from week six. Animals were humanely euthanized by neck dislocation, according to the specifications of the United Kingdom Animals (Scientific Procedures) Act, 1986 (ASPA), when they were 22 weeks old. The procedure was performed first time in the morning.

Cerebrum, cerebellum, hypothalamus, eyes, kidneys, spleen, liver, white adipose tissue (WAT), muscle, heart, intestinal sections (duodenum, jejunum, ileum, proximal colon, mid colon and distal colon), urine and blood were aseptically collected and immediately frozen in liquid nitrogen to be later on kept at − 80 °C until the day of the analysis. NMR sample preparation is detailed in S1.

### NMR analysis

^1^H NMR spectra from all biofluids and extracts, except the liver, were acquired on a Bruker Avance HD 700 MHz (Bruker BioSpin, Rheinstetten, Germany) with a TCI Cryoprobe and equipped with a cooled SampleJet sample changer from the same manufacturer. For liver samples, NMR spectra were acquired on a Bruker Avance III 500 MHz NMR spectrometer (Bruker BioSpin, Rheinstetten, Germany) equipped with a High-Resolution Magic Angle Spinning ^1^H NMR probe from the same manufacturer at a rotational speed of 5000 Hz.

For each one-dimensional (1D) NMR spectrum (for each tissue), a total of 64 scans were accumulated into 64 K data points with a spectral width of 13 ppm. Two types of 1D experiments were recorded, using standard pulse sequence: Carr–Purcell–Meiboom–Gill (CPMG, cpmgpr1d) (Meiboom and Gill 1958) and 1D NOESY (noesypr1d), both with water suppression applied during the relaxation time for 3 s. The mixing time of the noesypr1d was 50 ms in the case of the 500 MHz, and 10 ms in the case of the 700 MHz. The CPMG T_2_ filter was set at 39 ms. Additionally, one Correlation Spectroscopy (^1^H–^1^H COSY) was acquired on a selected representative sample from each bio-fluid and liver (Aue et al. 1975).

### Data processing and statistical analysis

Descriptive statistics and one-way ANOVA (factor = genetic background) were carried out on body weight, body weight gain (body weight *i* at time x − body weight *i* at time 0), liver and WAT relative weight (tissue weight i/body weight i) using RStudio (version 0.99.489—© 2009–2015 RStudio, Inc).

Spectra were pre-processed using MestReNova version 11.0.2-18153 (Mestrelab Research S.L., Spain) with manual phasing followed by automatic baseline correction using the Whittaker smoother algorithm and manual multipoint baseline correction when appropriate. Chemical shift calibrations were carried out relative to TSP (δ 0.00) for all tissues, except for liver and plasma where the glucose anomeric peak (δ 5.223) was used. NMR spectra were imported into Matlab version R2015b (Mathworks, UK) and analysed using the statistic toolbox and algorithms provided by Korrigan Toolbox version 0.1 (Korrigan Sciences Ltd., U.K.). In Matlab, residual water (δ 4.70–5.10) and noise (regions before δ 0.5 and after δ 9.5) were removed prior to matrix normalisation using a median-based probabilistic quotient method (Dieterle et al. 2006), except for plasma. The statistical strategy adopted for the analysis of the samples involved a preliminary unsupervised Principal Component Analysis (PCA), followed by a supervised pairwise Orthogonal Projection to Latent Structures Discriminant Analysis (O-PLS DA) (Bylesjö et al. 2006; Cloarec et al. 2005), which allowed the identification of specific modulations driven by T2D metabolic impairments. O-PLS DA models were evaluated for goodness of prediction (*Q*^*2*^Y value) using 7-fold cross-validation. Random permutation testing (300 randomisations) was then applied to validate the models and calculate a *p* value, which is the probability of obtaining such model purely by chance. Aliphatic and aromatic regions from urine datasets, where glucose signal is not present, were further studied applying a normalisation under total area (Dieterle et al. 2006) and interrogated by O-PLS DA model as described above. Metabolite identification was done using Chenomx NMR Suite 8.2 from Chenomx Inc (Edmonton, Canada), online publicly available databases: the Human Metabolome Data Base (HMDB, http://www.hmdb.ca), the Biological Magnetic Resonance data bank (BMRB, http://www.bmrb.wisc.edu) and published literature (Claus et al. 2011, 2008, Mora-Ortiz et al. 2019). A heatmap was calculated in R using the metabolites relative modulations (i.e. increase or decrease of the metabolite amongst diabetic individuals compared to control ones) obtained from the O-PLS DA analysis. The dendograms were calculated as part of the heatmap() function, and clustering was done calculating the mean of rows and columns.

## Results and discussion

### Body weight gain, and relative WAT weight was higher in diabetic individuals

**Fig. 1:**
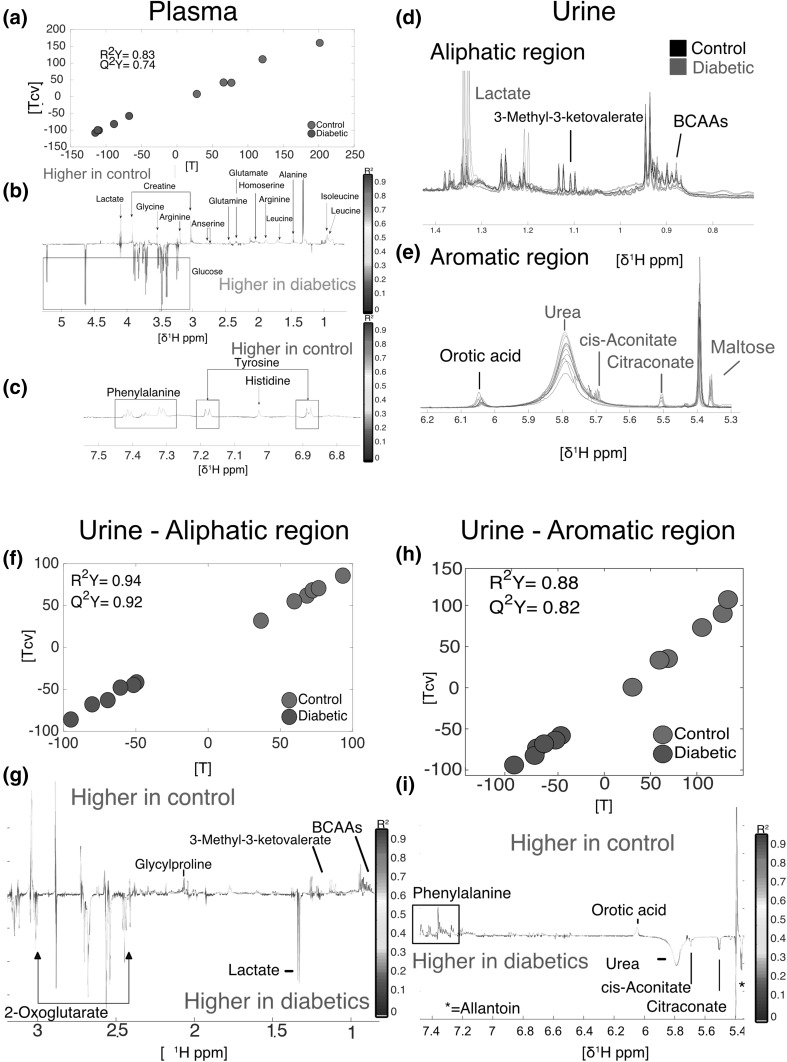
Metabolic differences in plasma (**a**, **b** and **c**) and urine (**d**, **e**, **f**, **g**, **h** and **i**). **a** plasma O-PLS DA model score plot calculated using all spectra as a matrix of independent variables and genetic background as predictor (R^2^Y = 0.83, Q^2^Y = 0.74, n = 10). **d** and **e** Aliphatic and aromatic regions of urine spectra showing differences between diabetic (red) and control (black) individuals. **f** and **g** O-PLS DA model score and loading plots calculated using the aliphatic (R^2^Y = 0.94, Q^2^Y = 0.92) region of urine. **h** and **i** O-PLS DA model calculated using the aromatic (R^2^Y = 0.88, Q^2^Y = 0.82) region of urine

The twelve animals of the study arrived at week four of age and were monitored until they were just over 22 weeks old, when they were euthanized. Body weight gain was significantly higher in diabetic animals (*p *< 0.01) (S2_Fig. 1a), in particular during the first 6 weeks (*p *< 0.001), when animals were between 5 and 11 weeks old and increased body weight gain more rapidly and variability was smaller. During the last week, diabetic individuals had 210% higher body weight gain than controls. WAT weight was significantly higher (434.8%) in the diabetic group (*p *< 0.001) (S2_Fig. 1b).

#### Biomarkers of T2D in biofluids

*Plasma* from diabetic individuals showed an increase in glucose and a decrease in alanine, anserine, arginine, creatine, glutamate, glutamine, glycine, histidine, homoserine, isoleucine, lactate, leucine, phenylalanine and tyrosine (Fig. 1a, b and c) (R^2^Y = 0.83, Q^2^Y = 0.74, n = 10). Leucine decrease was consistent with previous observations showing that ketogenesis is altered in the *db/db* mouse model. In addition, it has also been reported that BCAAs decreased in the late stages of the disease, which is consistent with the 22 weeks of age of the animals used in this work, effectively corresponding to a well-established disease (Kim et al. 2016; Li et al. 2015; Kim et al. 2016). This decrease in glucogenic and ketogenic amino acids among diabetic individuals is likely the result of a deficient intake of glucose by insulin-resistant cells, compensated by gluconeogenesis and ketogenesis from available amino acids, which is a well-known feature of human T2D (Menni et al. 2013). The impaired intake of glucose promotes gluconeogenesis in the liver which uses glucogenic amino acids as a fuel to produce pyruvate and 3-phosphoglycerate (Altmaier et al. 2008; Magnusson et al. 1992). These metabolic changes involving lactate and glucose pathway modulations go in accordance with the metabolomics changes previously described in plasma of animal models and patients in the literature (Nagana Gowda et al. 2008; Major et al. 2006).

The *urine* metabolic profile was characterised by an increase in glucose signal in the diabetic group dominating other metabolic changes. We therefore conducted a more focussed statistical analysis on the aliphatic and aromatic regions where glucose resonance is absent, as described in materials and methods. This allowed the identification of other metabolites, including 2-oxoglutarate, allantoin, cis-aconitate, citraconate, lactate and urea, which were increased in diabetic individuals. Conversely, 3-methyl-3-ketovalerate, BCAAs, glycylproline, orotic acid and phenylalanine had lower levels in diabetic individuals (Fig. 1, panels d, e, g and i). Glucose set aside, the most noticeable differences were the presence of high citraconate in diabetic individuals, which were not detected in controls. This metabolite is an isomeric carboxylic acid, derived from citrate which is known to inhibit fumarate reduction (Vaidyanathan et al. 2001; Hao et al. 2017). As a consequence, this would slow down the rest of the Krebs cycle and therefore limit the use of acetylCoA to produce ATP (You et al. 2016; Hao et al. 2017). Eventually, excessive acetylCoA may be directed towards de novo lipid synthesis and contribute to lipid accumulation in the liver (Solinas et al. 2015; Postic and Girard 2008). Conversely, 2-keto-3-methylvalerate, an intermediate of the degradation of isoleucine, was significantly decreased in diabetics, consistent with observed lower levels of BCAAs in plasma.

#### Biomarkers of T2D in muscles and major metabolic organs

**Fig. 2:**
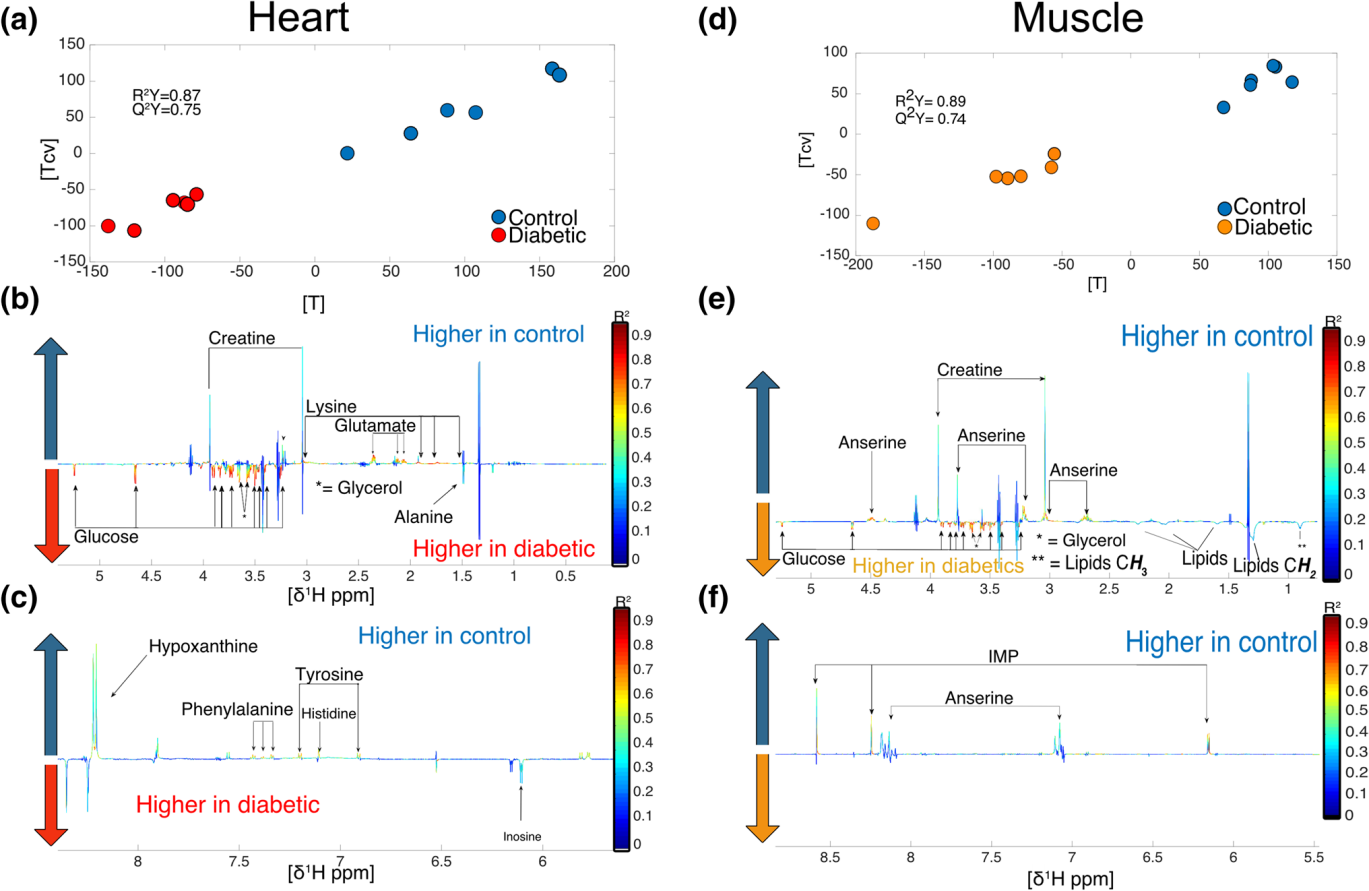
Metabolomics differences in heart and muscle. **a** heart O-PLS DA model (R^2^Y = 0.87, Q^2^Y = 0.75) score plot calculated using all spectra as a matrix (n = 12) of independent variables and genetic background as predictor. **b** and **c** loading plots from the heart O-PLS DA model. **d** score plot of the muscle O-PLS DA model calculated using all spectra as a matrix (n = 12) of independent variables and genetic background as predictor (R^2^Y = 0.89, Q^2^Y = 0.74). **e** and **f** loading plots from the O-PLS DA model carried out in muscle samples

*Heart* from diabetic individuals had higher levels of alanine, glucose, glycerol and inosine and lower levels of creatine, glutamate, histidine, hypoxanthine, lysine phenylalanine and tyrosine (Fig. 2, panels a, b and c) (R^2^Y = 0.87, Q^2^Y = 0.76). The O-PLS DA analysis of skeletal *muscle* identified higher levels of glucose, glycerol and lipids and lower levels of anserine, creatine and IMP in diabetic individuals (R^2^Y = 0.89, Q^2^Y = 0.74) (Fig. 2, panels d, e and f). Anserine acts as a buffer in muscle tissues, and is essential for good functioning. In particular, it protects against protein trans-glycation, which is the first step of advanced glycation end products (AGEs) known to trigger a number of physiopathologic processes (Boldyrev et al. 2013; Fournet et al. 2018). Thus, a reduction in muscular anserine may be an unexplored mechanism contributing to the physiopathology of T2D.

**Fig. 3:**
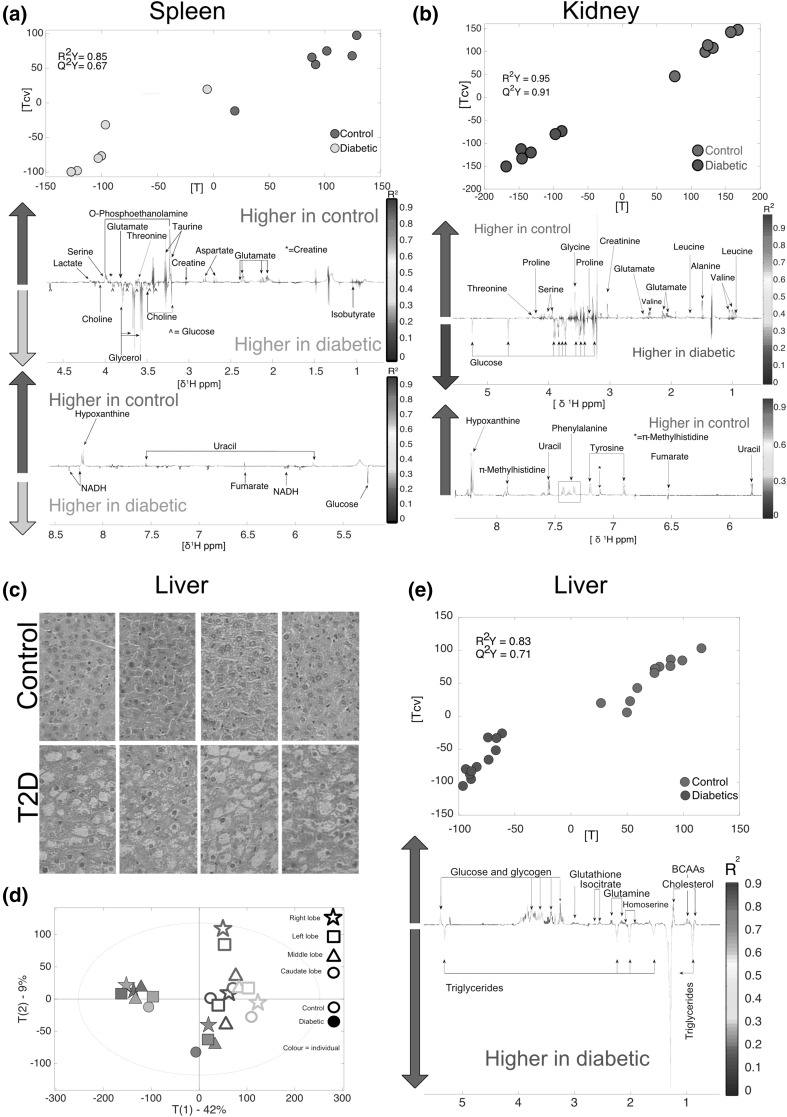
Metabolomics and histological analysis of liver and metabolomics analysis of spleen and kidney. **a** Liver histology, type 2 diabetes individuals showed clear fat accumulation characteristic of steatosis (lower row) compared to control liver (top row) in all the liver lobes. **b** PCA showing clusters control and diabetic individuals respectively. Higher variability was observed in healthy individuals. **c** O-PLS DA model calculated using all liver spectra as a matrix of independent values (R^2^Y = 0.83 and Q^2^Y = 0.71). **d** spleen O-PLS DA model calculated using all spectra as a matrix (n = 12) of independent variables and genetic background as predictor (R^2^Y = 0.85, Q^2^Y = 0.67, n = 12). **e** Kidney O-PLS DA model calculated using all spectra as a matrix (n = 12) of independent variables and genetic background as predictor (R^2^Y = 0.95, Q^2^Y = 0.91)

The *spleen* O-PLS DA (R^2^Y = 0.85, Q^2^Y = 0.67, n = 12) identified that diabetic individuals had higher levels of choline, fumarate, glucose, glycerol, isobutyrate and NADH. Conversely, diabetic individuals had lower levels of aspartate, creatine, glutamate, hypoxanthine, lactate, *O*-phosphoethanolamine, serine, taurine, threonine and uracil (Fig. 3a).

The O-PLS DA conducted on *kidney* samples (R^2^Y = 0.95, Q^2^Y = 0.91) allowed the identification of metabolites differing between diabetic and control individuals (Fig. 3b). Diabetic individuals had higher levels of glucose and lower levels of alanine, creatinine, fumarate, glutamate, glycine, hypoxanthine, leucine, *Π*-methylhistidine, phenylalanine, proline, serine, threonine, tyrosine, uracil and valine.

*Heart*, *spleen* and *kidney* followed a similar pattern to what was observed in plasma, where glucogenic and ketogenic amino-acids were decreased among diabetic individuals, consistent with an activation of the gluconeogenesis pathway. Glucose levels were increased among diabetic individuals in these tissues.

Diabetic nephropathy (DN) is a leading cause of death and one of the major reasons of end stage renal disease (Shao et al. 2013; Shaw et al. 2010); yet, metabolic characterisation of changes occurring in DN remain unresolved and urinary tests fail to give an accurate early diagnosis (Wei et al. 2015; Shao et al. 2013). ^1^H-NMR metabolomics analysis identified sixteen metabolites that were modulated in the kidneys of diabetic individuals. Likewise, many intermediates involved in the TCA cycle and glycolysis were decreased in diabetic individuals, while glucose was increased. Similar changes were previously reported when comparing *db/db* versus *db/*+ individuals in metabolomics studies using targeted Liquid Chromatography-Mass Spectroscopy (LC/MS), Gas Chromatography-Mass Spectroscopy (GC/MS) (Sas et al. 2016) and ^1^H-NMR metabolomics (Wei et al. 2015). Kidneys displayed a metabolic impairment very similar to that observed in the spleen (Fig. 3a and b). Similarly, sixteen metabolites were modified in the spleen. Metabolic changes in the spleen are very complex and reflect a complete shift in metabolism characterised by excessive NADH production, which is one of the main molecular features of the diabetic phenotype due to excessive glycolysis (Wu et al. 2016). One of the main differences observed in the spleen compared to the kidney, were decreased amounts of *O*-phosphoethanolamine in diabetic individuals. *O*-phosphoethanolamine plays an important role in sphingolipid metabolism in mammals. This is the only pathway that transforms sphingolipids to non-sphingolipids through sphingosine-1-phosphate lyase (Frolkis et al. 2010). Therefore, future efforts should focus on the pathways associated with these biomarkers.

Contrarily to what was observed in the spleen and the kidney, the *heart* tissue, which has traditionally received more attention due to the cardiovascular complications associated with T2D, only presented a few metabolic modulations: alanine, glucose, glycerol and inosine were increased in diabetic individuals, while creatine, glutamate, hypoxanthine and lysine were decreased.

Interestingly, de Castro et al. (2013) also observed changes in creatine in the cardiac tissue in the rat Zucker *fa/fa* model. Dysfunctionality of the creatine kinase system happens from an early stage of diabetic impairment in human hearts but has not been associated with ventricular dysfunction (Scheuermann-Freestone et al. 2003; Kouzu et al. 2015). Creatine has been suggested as a potential supplement to improve glucose tolerance and seemed promising when combined with exercise (Gualano et al. 2007; Gualano et al. 2011). Other studies have shown that creatinine mitigated hyperglycaemia and reduced the insulinogenic index in rodents, thus delaying the initiation of diabetes, and helped muscle recovery in both rats and humans (Ferrante et al. 2000; Op’t Eijnde et al. 2006).

*Liver* histology showed a clear pattern of fat accumulation characteristic of steatosis in diabetic livers (Fig. 3c). It was not possible to identify metabolic differences between lobes, but healthy individuals showed higher inter-individual variability (Fig. 3d). Liver O-PLS DA analysis (R^2^Y = 0.83, Q^2^Y = 0.71) were driven by higher levels of triglycerides in diabetic individuals while minor changes in polar metabolites were also observed (Fig. 3e) Changes in polar metabolites were not consistent with previous findings in the rat *fa/fa* model (Claus et al. 2011). However, different NMR-based techniques were used to measure the hepatic metabolic fingerprints in the two studies and the results are therefore difficult to compare. Yet, high levels of triglycerides is a characteristic feature of the diabetic liver, and has previously been associated with fatty liver (Sakitani et al. 2017), which is also evidenced by the histological results obtained in this analysis. Non-Alcoholic Fatty Liver Disease (NAFLD) is the major cause leading to cirrhosis (Hazlehurst et al. 2016; Bugianesi et al. 2007), which increases by 75% the risk of developing liver cancer (Bhatt and Smith 2015; Zawdie et al. 2018). The *db/db* mouse model may therefore represent a suitable experimental model to study the evolution of early hepatic metabolic changes associated with NAFLD progression in T2D.

Small and large intestine showed a lower number of metabolomics modulations compared to the numerous changes observed in major metabolic organs (S3).

#### Biomarkers of T2D in the brain

**Fig. 4:**
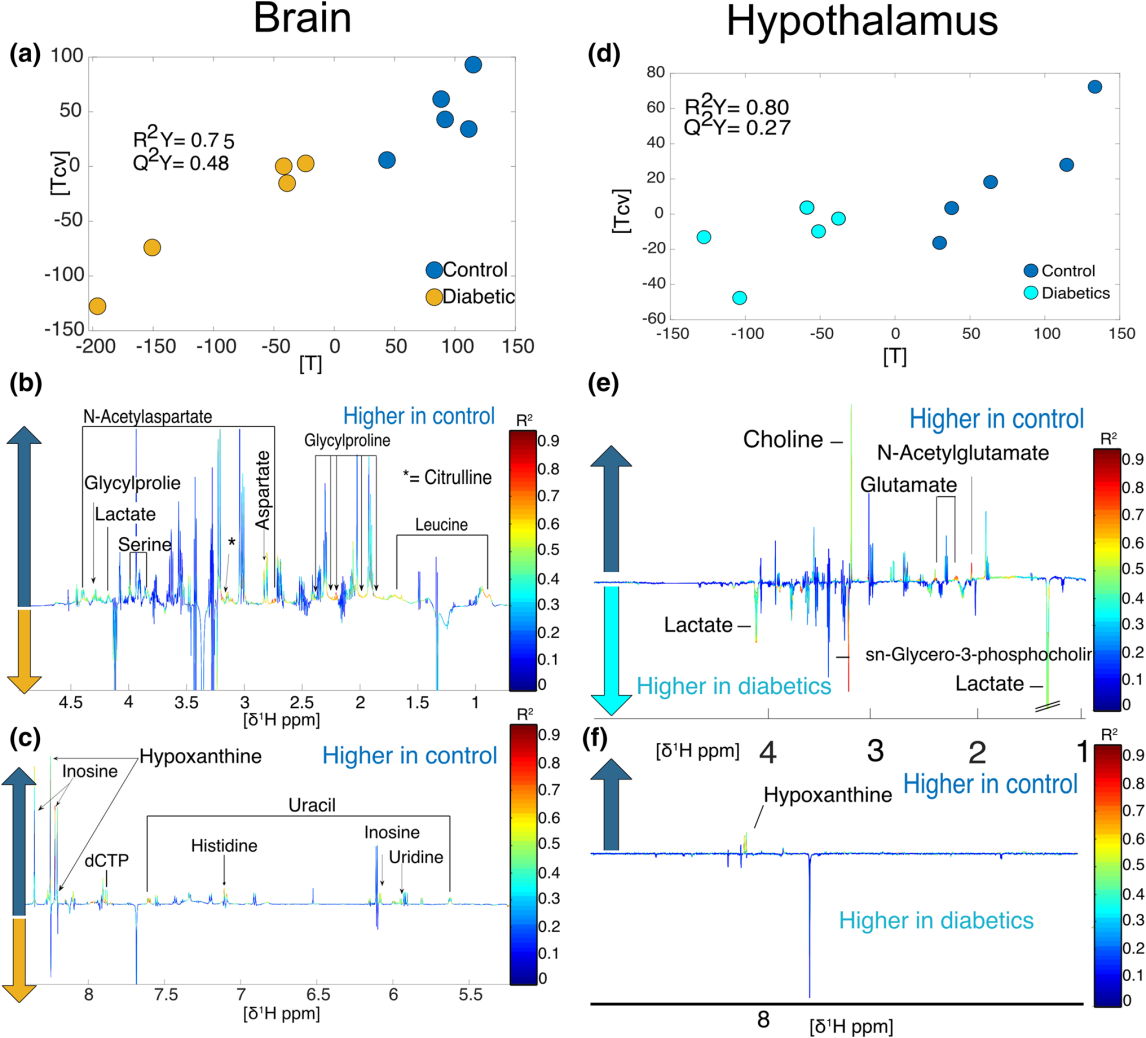
Metabolomics analysis of cerebrum and hypothalamus. **a** brain O-PLS DA model score plot calculated using all spectra as matrix (n = 10) of independent variables and genetic background as predictor (R^2^Y = 0.75, Q^2^Y = 0.48). **b** and **c** loadings for brain O-PLS DA model. **d** hypothalamus O-PLS DA model (R^2^Y = 0.80, Q^2^Y = 0.27). **e** and **f** loadings for hypothalamus O-PLS DA model

The analysis of the metabolic profile of *cerebrum* (R^2^Y = 0.75, Q^2^Y = 0.48, n = 10, Fig. 4a) detected several compounds decreased in the diabetic mouse including aspartate, citrulline, dCTP, glycylproline, histidine, hypoxanthine, inosine, lactate, leucine, *N*-acetylaspartate, serine, uracil and uridine (Fig. 4b and c). Leucine is an essential amino acid and is currently considered one of the most important BCAAs in brain metabolism. Brain amino acids are used to maintain low intra-synaptic concentrations of glutamic acid, an excitatory neurotransmitter, to maximize the signal-to-noise ratio when it is released from nerve terminals (Meldrum 2000). In this way, the potential excitotoxicity of glutamatergic stimulation is kept to a minimum (Yudkoff et al. 2005; Nicholls et al. 1999). Hence, leucine easily penetrates the brain, promoting buffering mechanisms to maintain glutamate in optimum concentrations (Oldendorf 1971; Smith et al. 1987). Lower concentrations of leucine in diabetic individuals may therefore indicate a failure in the regulation of neurotransmitters.

In the *hypothalamus*, although the metabolic effects of diabetes were not as strong as in the cerebrum, as indicated by a lower goodness of prediction (Q^2^Y = 0.27), it was still possible to identify some metabolites that increased amongst healthy individuals, including choline, glutamate, hypoxanthine and *N*-acetylglutamate. By contrast, diabetic individuals were associated with higher levels of lactate and *sn*-glycero-3-phosphocholine (Fig. 4, panels d, e and f, R^2^Y = 0.80, Q^2^Y = 0.27). Neurotransmission in the ventromedial hypothalamus is mediated by GABAergic neurotransmission. The suppression of GABAergic neurotransmission is necessary to activate the counter-regulatory responses to hypoglycemia (Chan et al. 2006; Zhu et al. 2010). Lactate contributes to counter-regulatory failure in hypoglycemic diabetic patients. This is carried out by increasing ventromedial hypothalamus GABA levels (Chan et al. 2013). Glutamate, glutamine and GABA were also reduced in the eye, which suggests that the GABA pathway is also altered in diabetic retinopathies. In previous studies, it has been shown that GABA content and activity of glutamate decarboxylase (GAD) and GABA transaminase (GABA-T) in the retina of diabetic STZ-treated rats was decreased, which has also been reported in the *db/db* mouse model (Honda et al. 1998; Ishikawa et al. 1996; Kobayashi et al. 1999). GABA content and GAD activity were reduced in the superior colliculus of STZ-treated rats. Altogether this indicates that GABA metabolism is altered in diabetic individuals.

Serine hypothalamic levels were lower among diabetic individuals. Serine deficiency resulting from a defect in biosynthesis is well documented. Three main causes are known: (i) 3-phosphoglycerate dehydrogenase deficiency, (ii) 3-phosphoserine phosphatase deficiency and (iii) phosphoserine aminotransferase deficiency. These enzyme defects result in severe psychomotor retardation and microcephaly (Singh and Singh 2011; Madeira et al. 2015). This suggests that some of the motor difficulties observed in the *db/db* mouse model could be linked to decreased serine levels in cerebrum, in addition to excessive body weight and loss of muscle mass.

No differences between diabetic and control individuals were found in the *cerebellum*.

Interestingly, the *eye* presented one of the most distinctive metabolic features, characterized by increased glucose and lipid levels, and reduced levels of alanine, citrulline, GABA, glutamate, glutamine, histidine, hypoxanthine, inosine, isocitrate, *myo*-inositol, *O*-phosphocholine, phenylalanine and tyrosine (R^2^Y = 0.81, Q^2^Y = 0.67, S4). Diabetic retinopathy was previously linked to an increased activity of polyol synthesis pathway (Lorenzi 2007; Gabbay 1973). As a consequence, reduced levels of *myo*-inositol are expected and have indeed been observed in the eyes of diabetic rabbits and rats (Loy et al. 1990; Gabbay 1973). In our *db/db* mouse model, *myo*-inositol was also decreased amongst diabetic individuals. It has been previously reported that treating STZ-induced diabetic rats with *myo*-inositol was an effective method to avoid metabolic impairments associated with activation of the polyol pathway (Coppey et al. 2002). Findings in the *db/db* model are consistent with the literature and indicate that this could be a valid model for the development of new therapies to maintain adequate levels of *myo*-inositol in T2D.

Other metabolomics changes in the eye affected citrulline levels. Nitric oxide (NO) is produced when *L*-arginine is transformed to *L*-citrulline by the enzymatic activity of NO synthase (NOS) (Bredt and Snyder 1994). It has been shown that during the onset of diabetic retinopathy in STZ-treated rat retinas, T2D damages the functioning of the nNOS-positive amacrine cells and reduces NO generation via nNOS (Goto et al. 2005). A similar process to what was observed in STZ-treated rats may occur in the diabetic mouse model BKS.Cg-Dock7 < m > +/+ Lepr < db >/J. For further information, the *p*-values resulted from the permutations carried out in every model can be found in S5.

In total, 61 distinct metabolites were identified associated with diabetic modulations. Glucose and glutamate were the most commonly associated metabolites, and they were significantly increased across nine biological matrices. Kidneys, spleen, eye and plasma, clustering all in the same super group in the heatmap (S6), were the organs and fluids that displayed the most varied metabolic changes. This clustering was partially due to a decrease in amino acids. The large heterogeneity in the metabolic response that is strongly organ-specific prevented further grouping of the organs.

**Fig. 5:**
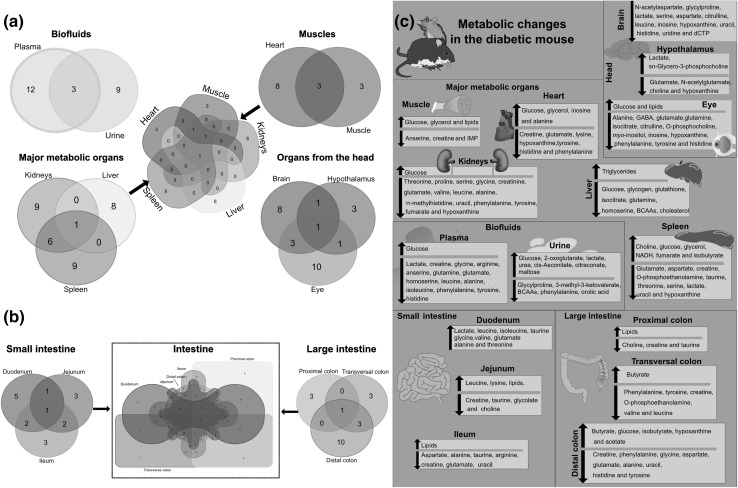
Left panels: Venn diagrams showing number of metabolites shared by different organs following the classification adopted in the study. **a** metabolites shared by main organs and biofluids. **b** metabolites in common along different sections of the small and large intestine. Right panels: Tissue-specific summary of the metabolic impairments associated with type 2 diabetes in the *db/db* mouse model BKS.Cg-Dock7 < m > +/+ Lepr < db >/J

In total, 16 metabolites were found modulated in kidney and spleen, and 15 in eye and plasma. Table 1 and Fig. 5 and S6 summarize these findings. Out of the 15-metabolic modulations detected in the kidney and the spleen, 6 were shared by these two organs (Fig. 5a). This highlights the need to devote more attention to the role of kidneys and spleen in T2D. Moreover, metabolic modulations showed that both, proximal and distal colon were affected by changes in tyrosine and phenylalanine, whose availability in these biological matrices is strongly influenced by the gut microbiota (S6, Dodd et al. 2017; Fujisaka et al. 2018). These modulations were also present in plasma, heart, eye and kidney. This suggests that further studies should investigate the potential influence of the gut microbiota on the amino acid imbalance associated with T2D.

**Table 1:** Sixty-one metabolites were found associated with metabolic impairment modulations related to Type 2 Diabetes

	Metabolite	Decreased	Increased	Peaks (ppm shift)
1	Acetate	N/A	Distal colon	CH_3_ 1.92 s
2	Alanine	Kidneys, eye, plasma, ileum, distal colon	Heart, duodenum	βCH_3_ 1.46 d, αCH 3.78 q
3	Anserine	Muscle, plasma	N/A	βCH_2_ 2.68 m, ½ δCH_2_ 3.03 dd, ½ δCH_2_ 3.21 dd, αCH_2_ 3.22 m, CH_3_ 3.76 s, γCH_2_ 4.48 m, CH 7.07 s, N–CH 8.20 s
4	Arginine	Plasma, ileum	N/A	γCH_2_ 1.66 m, βCH_2_ 1.91 m, δCH_2_ 3.27 t, αCH 3.77 t
5	Aspartate	Cerebrum, spleen, ileum, distal colon	N/A	½ βCH_2_ 2.68 dd, ½βCH_2_ 2.82 dd, αCH 3.91 dd
6	BCAAs	Liver, urine	N/A	See leucine, isoleucine and valine
7	Butyrate	N/A	Transversal colon, distal colon	CH_3_ 0.88 t, βCH_2_ 1.55 m, αCH_2_ 2.15 t
8	Cholesterol	Liver	N/A	CH _3_(CH_2_)n 0.84 t, (CH _2_)n 1.25 m, CH_2_–C=C 2.04 m
9	Choline	Hypothalamus, jejunum, proximal colon	Spleen	N–(CH_3_)_3_ 3.22 s, βCH_2_ 3.53 dd, αCH_2_ 4.06 t
10	*cis*-Aconitate	N/A	Urine	CH 5.71 s, CH_2_ 3.11 s
11	Citraconate	N/A	Urine	CH 5.51 s, CH_3_ 1.91 s
12	Citrulline	Cerebrum, eye	N/A	δCH_2_ 3.15 q, βCH_2_ 1.86 m, γCH2 1.57 m
13	Creatine	Muscle, spleen, heart, plasma, jejunum, ileum, proximal, transversal and distal colon	N/A	N–CH_3_ 3.03 s, N–CH_2_ 3.94 s
14	Creatinine	Kidneys	N/A	N–CH_3_ 3.05 s, N–CH_2_ 4.06 s
15	dCTP	Cerebrum	N/A	N–CH 7.89 d, C=CH 6.31 d, CH 6.11 d, CH 4.72 t, CH 4.58 t, CH_2_ 4.22 d, CH 4.20 d
16	Fumarate	Kidney	Spleen	HCOOH 6.51 s
17	GABA	Eye		βCH_2_ 1.88 m, αCH_2_ 2.29 t, γCH_2_ 3.01 t
18	Glucose	Liver	Kidneys, muscle, eye, heart, spleen, plasma, distal colon, urine	C_4_H 3.42 m, C_2_H 3.54 m, CH3 3.72 m, ½ C_6_H_2_ 3.73 m, ½C_6_H_2_ 3.77 m, C_5_H 3.87 m, C_1_H 5.23 d
19	Glutamate	Kidneys, eye, hypothalamus, spleen, heart, plasma, ileum, distal colon	Duodenum	βCH_2_ 2.02 m, γCH_2_ 2.34 m, αCH 3.76 dd
20	Glutamine	Eye, liver, plasma	N/A	βCH_2_ 2.15 m, γCH_2_ 2.44 m, αCH 3.77 t
21	Glutathione	Liver	N/A	CH_2_ 2.17 m, CH_2_ 2.53 m, S–CH_2_ 2.95 dd, N–CH 3.83 m, CH 4.56 q
22	Glycerol	N/A	Muscle, spleen, heart	½ CH_2_ 3.58 m, ½CH_2_ 3.62 m, CH 3.77 t
23	Glycogen	Liver	N/A	C_2_H 3.63 dd, C_4_H 3.66 dd, C5H 3.83 q, C_6_H 3.87 d, C_3_H 3.98 d, C1H 5.41 m
24	Glycine	Kidneys, plasma, distal colon	Duodenum	αCH_2_ 3.55 s
25	Glycolate	Jejunum	N/A	C_2_H 3.9 s
26	Glycylproline	Cerebrum, urine	N/A	½ O=C–CH 4.29 m, ½ O=C–CH 4.26 m, ½ H_2_N–CH_2_ 3.94 s, ¼ H_2_N–CH_2_ 3.89 d, ¼ H_2_N–CH_2_ 3.63 d, N–CH_2_ 3.57 m, NC–CH_2_ 2.18 m, 2.28 m, 2.13 m, 1.99 m, 1.97 m, NC–CH_2_ 1.92 m
27	Histidine	Cerebrum, eye, heart, plasma, distal colon	N/A	½ CH_2_ 3.16 dd, ½ CH_2_ 3.23 dd, CH 3.98 dd, CH 7.09 s, CH 7.90 s
28	Homoserine	Plasma, liver	N/A	N–CH 3.85 dd, O–CH_2_ 3.77 m, ½CH2 2.14 m, ½CH_2_ 2.01 m
29	Hypoxanthine	Kidneys, cerebrum, hypothalamus, eye, spleen, heart	Distal colon	CH 8.18 s, CH 8.21 s
30	IMP	Muscle	N/A	N=(CH)–N 8.56 s, N=(CH)–NH 8.22 s, N–(CH)–O 6.13 d, HO–CH 4.50 m, NCO–CH 4.36 m, O=PO–CH_2_ 4.02 m
31	Inosine	Cerebrum, eye	Heart	½ CH2 3.83 dd, ½ CH_2_ 3.91 dd, C1H 4.27 dd, C_2_H 4.43 dd, C_3_H 4.76 t, C_4_H 6.09 d, NH–CH 8.23 s, N–CH 8.34 s
32	Isobutyrate	N/A	Spleen, distal colon	(CH_3_)_2_ 1.05 d, CH 2.38 m
33	Isocitrate	Eye, liver	N/A	CH 4.05 d, CH 2.99 m, CH_2_ 2.48 dq
34	Isoleucine	Plasma	Duodenum	γCH_3_ 0.94 t, δCH_3_ 1.02 d, ½ γCH_2_ 1.26 m, ½ γCH_2_1.47 ddd, βCH 2.01 m, αCH 3.65 d
35	Lactate	Cerebrum, spleen, plasma	Hypothalamus, duodenum, urine	βCH_3_ 1.33 d, αCH 4.12 q
36	Leucine	Kidneys, cerebrum, plasma, transversal colon	Duodenum, jejunum	δCH_3_ 0.93 d, βCH_2_ 0.94 d, γCH 1.71 m, αCH 3.73 m
37	Lipids	N/A	Muscle, eye, jejunum, ileum, proximal colon	N/A
38	Lysine	Heart	Jejunum	γCH_2_ 1.46 m, δCH_2_ 1.71 m, βCH_2_ 1.84 m, εCH_2_ 3.01 t
39	Maltose	Urine	N/A	O–(CH)–O 5.4 d, O–(CH)–OH 5.22 d, ½OCH–(CH)–OH 3.96 m, ½ CH_2_ 3.9 dd, O–(CH)–CHO 3.9 dd, CH_2_ 3.84 m, ½CH_2_ 3.76 m, ½ OCH–(CH)–OH 3.76 m, O–(CH)–CH_2_OH 3.7 m, HO–CH 3.66 m, O–(CH)–CHO 3.62 m, OCH–(CH)–OH 3.58 m, O–(CH)–CH_2_OH 3.58 m, HO–CH 3.41 t, HO–CH 3.27 dd
40	*∏*-Methylhistidine	Kidney	N/A	N–CH 8.10 s, N=CH 7.12 s, NH_2_–CH 3.96 dd, N–CH_3_ 3.74 s, ½CH_2_ 3.31 dd, ½CH_2_ 3.22 dd
41	*Myo*-Inositol	Eye	N/A	C5H 3.29 t, C1H C_3_H 3.53 dd, C4H C_5_H 3.63 t, C2H 4.06 t
42	*N*-Acetylaspartate	Cerebrum	N/A	NH 7.94 d, CH 4.38 ddd, ½CH_2_ 2.68 dd, ½CH_2_ 2.49 d, CH_3_ 2.01 s
43	*N*-Acetylglutamate	Hypothalamus	N/A	NH 7.97 d, N–CH 4.10 m, O=C–CH_2_ 2.22 t, ½ CH_2_ 2.05 m, O=C–CH_3_ 2.02 s, ½ CH_2_ 1.86 m
44	NADH	N/A	Spleen	N=(CH)–N–C 8.46 s, N=(CH)–N=C 8.23 s, N–(CH)=C 6.94 s, O–(CH)–N 6.12 d, N–(CH)=C 5.97 dd, O–(CH)–N 4.78 m, C–(CH)=C 4.78 m, HO–CH 4.70 m, HO–CH 4.49 t, O–CH 4.36 s, ½ P–O–CH_2_ 4.25 m, ½ C–(CH)–C 4.25 m, ½ O–CH 4.25 m, ½ P–O–CH_2_ 4.08 m, ½ C–(CH)–C 4.08 m, ½ O–CH 4.08 m, C=C–CH_2_ 2.70 m
45	*O*-Phosphocholine	Eye	N/A	N–(CH_3_)_3_ 3.21 s, CH_2_ 3.58 m, O–CH_2_ 4.16 m
46	*O*-Phosphoethanolamine	Spleen, transversal colon	N/A	CH_2_ 4.0 td, CH_2_ 3.2 t
47	Orotic acid	Urine	N/A	CH 6.18 s
48	Phenylalanine	Kidneys, eye, heart, plasma, transversal colon, distal colon, urine	N/A	½ βCH_2_ 3.12 dd, ½ βCH_2_ 3.26 dd, C_3_H C_5_H 7.33 m, C_4_H 7.35 m, C_3_H C_6_H 7.40 m
49	Proline	Kidneys	N/A	γCH_2_ 2.03 m, ½βCH_2_ 2.03 m, ½βCH_2_ 3.35 m, ½δCH_2_ 3.38 m, ½δCH_2_ 3.41 m, αCH 4.41 dd
50	Serine	Kidneys, spleen, cerebrum	N/A	αCH 3.85 dd, ½βCH_2_ 3.95 dd, ½βCH_2_ 3.95 dd
51	*Sn*-glycero-3-phosphocholine	N/A	Hypothalamus	O=PO–CH_2_ 4.3 m, O=PO–CH_2_ 3.9 m, HO–CH 3.9 m, HO–CH_2_ 3.6 m, N–CH_2_ 3.6 m, N–(CH_3_)_3_ 3.2 s
52	Taurine	Spleen, jejunum, ileum, proximal colon	Duodenum	N–CH_2_ 3.26 t, S–CH_2_ 3.43 t
53	Threonine	Kidneys, spleen	Duodenum	γCH_3_ 1.32 d, αCH 3.60 d, βCH 4.25 m
54	Triglycerides	N/A	Liver	C***H*** _3_CH_2_CH_2_C = 0.87 t, C***H*** _2_CH_2_CH_2_CO 1.29 m, C***H***_2_CH_2_O 1.57 m, C***H*** _2_–C=C 2.04 m, C***H*** _2_–C–O 2.24 m, =CH–C***H*** _2_–CH = 2.75 m, CH=CHC***H*** _2_5.32 m
55	Tyrosine	Eye, kidneys, heart, plasma, transversal colon, distal colon	N/A	½ CH_2_ 3.04 dd, ½CH_2_ 3.18 dd, N–CH 3.94 dd, C_3_H C_5_H 6.89 m, C_2_H C_6_H 7.18 m
56	Uracil	Kidneys, cerebrum, spleen, ileum, distal colon	N/A	C_5_H 5.80 d, C_6_H 7.54 d
57	Urea	N/A	Urine	NH_2_ br 5.80
58	Uridine	Cerebrum	N/A	½ CH_2_ 3.81 dd, ½CH_2_ 3.92 dd, C_4_H 4.12 dt, C_3_H 4.24 dd, C_2_H 4.36 dd, C_1_H 5.88 d, C_5_H 5.92 m, C_6_H 7.88 d
59	Valine	Kidneys, transversal colon	Duodenum	γCH_3_ 0.98 d, γ’CH_3_ 1.04 d, βCH 2.27 m, αCH 3.62 d
60	2-Oxoglutarate	N/A	Urine	βCH_2_ 3.01 t, γCH_2_ 2.44 t
61	3-Methyl-3-ketovalerate	Urine	N/A	CH 2.92 m, ½ CH_2_ 1.69 m, ½ CH_2_ 1.45 m, CH_3_ 1.09 d, CH_3_ 0.88 t

Peaks are pH sensitive

*N/A* not applicable, modulations were not found in that direction. Key: *s* singlet, *d* doublet, *t* triplet, *m* multiplet, *bs* broad singlet

## Conclusion

The present study reports qualitative differences in 16 tissues between diabetic *db/db* mouse model BKS.Cg-Dock7 < m > +/+ Lepr < db >/J and their wild type control, identifying over 60 metabolites modulated between these two groups. This study represents the most comprehensive tissue-specific metabolic characterization of this model and is intended to be used as a reference for further research in this area. Kidney, spleen, eye and plasma were the organs that showed the most metabolic modulations between control and diabetic individuals. In total, across all the tissues and biofluids studied, 61 biomarkers were found associated with diabetes.

Some limitations of this study included a restricted coverage of some potentially important metabolites, such as bile acids and lipids, due to the nature of the methods employed and further studies are necessary to uncover these modulations. The use of a small number of mice of both genders, which impeded a study of gender specific changes is another limitation. Future studies, should also consider the impact of diet and environment on the metabolic modulations associated with diabetes. Hence, diabetic studies should be addressed as part of an integrative approach considering metabolomics along other ‘omics’ technologies such as metagenomics.

## Electronic supplementary material

Below is the link to the electronic supplementary material.

Supplementary material 1 (DOCX 121 kb)

Supplementary material 2 (DOCX 234 kb)

Supplementary material 3 (DOCX 62161 kb)

Supplementary material 4 (DOCX 13453 kb)

Supplementary material 5 (XLSX 36 kb)

Supplementary material 6 (DOCX 250 kb)

Supplementary material 7 (ZIP 49544 kb)

## Abbreviations

NMR: Nuclear magnetic resonance
T2D: Type two diabetes
