# Supplementary material for: NMR metabolomics identifies over 60 biomarkers associated with Type II Diabetes impairment in db/db mice
Source: Metabolomics. 2019 Jun 10;15(6):89. doi: 10.1007/s11306-019-1548-8 (PMC6556514; doi:10.1007/s11306-019-1548-8)
Supplement: Supplementary file 1 — Supplementary material 1 (DOCX 121 kb) [file 11306_2019_1548_MOESM1_ESM.docx]

**Sample preparation:**

Thirty microliters of **urine** from each animal were collected and mixed with 30 µl of phosphate buffer made up in 80% D_2_O containing 0.1% deuterated 3(trimethylsilyl)propionic acid (TSP) (pH 7.4) before being placed in 1.7 mm capillary tubes for NMR analysis. Total **blood** samples were left to thaw at room temperature and centrifuged for 5 min at 2,000 g at 4°C. Samples of serum (30 μl) were collected. These samples were diluted in 150 μl of D_2_O. One hundred and eighty microliters were transferred into 3 mm NMR tubes and kept at 4°C until the samples were run in the NMR spectrometer.

Tissue biopsies from **muscle, heart, spleen, intestinal sections** and **kidney** acquisitions (~75 mg) were cut from frozen tissues on a cold surface to prevent defrosting. These samples were homogenised in a TissueLyser LT (Qiagen, Germany) with 1 mm diameter zirconia beads and 1 mL of methanol/water (1:1) for polar metabolite extraction and centrifuged at 5,000 g for 5 min at 4°C. The supernatants were collected and the methanol fraction evaporated in a speed vacuum concentrator (Eppendorf, Germany) at 45°C for 4 hour. Extracts were reconstituted in 250 μL of phosphate buffer and 200 μL were transferred into 3 mm NMR tubes. Samples were kept at 4°C in a refrigerated SampleJet (Bruker Biopsin, Rheinstetten, Germany) until they were run in the NMR spectrometer.

**Cerebrum** (~100 mg), **cerebellum** (~80 mg), **hypothalamus** (~20 mg) and **eye** (~30 mg) were cut from frozen tissues on a cold surface to prevent defrosting. These samples were homogenised in a TissueLyser LT (Qiagen, Germany) with 1 mm diameter zirconia beads and 0.5 mL of methanol/water (3:1) for polar metabolite extraction and 0.3 mL of chloroform, and centrifuged at 5,000 g for 10 min at 4°C. The supernatants were collected and the methanol fraction was evaporated in a speed vacuum concentrator (Eppendorf, Germany) at 45°C for 4 hour. Extracts were reconstituted in 250 μL and 200 μL were transferred into 3 mm NMR tubes. Samples were kept at 4°C in a SampleJet until NMR acquisition.

**WAT** (~100 mg) was cut from frozen in a cold surface to avoid degradation of the metabolites. The samples there were then homogenised in a TissueLyser LT (Qiagen, Germany) with 1 mm diameter zirconia beads and 0.5 mL of methanol/water (3:1). Consequently, the samples were centrifuged at 5,000 g for 15 minutes at 4°C. The supernatant was then evaporated in a vacuum concentrator (Eppendorf, Germany) at 45°C for 3 hours and reconstituted in 200 μL of phosphate buffer (0.2 M, pH 7.4) in deuterated water (D_2_0) with 0.1% of TSP (w/v). Then, this polar extract (or aqueous fraction) was transferred into 3 mm NMR tubes and kept into the fridge until the samples were analysed. The apolar pellet was reconstituted in 600 μL of chloroform/methanol (3:1) and centrifuged at 5,000 g for 5 minutes at 4°C, the supernatant was then collected and put into the speed vacuum (Eppendorf, Germany) at 45°C for 1 hour. The pellet was then suspended in 200 μL of deuterated chloroform with 0.05 TMS (tetramethylsilane) (v/v). Subsequently samples were then centrifuged at 5,000 g for 5 minutes and then 550 μL of this lipophilic fraction were transferred into 3 mm NMR tubes (Waters *et al.*, 2002). Samples were kept at 4°C in a SampleJet until NMR acquisition.

**Liver** samples (~25 mg) were cut from frozen in a cold surface to avoid degradation of the samples. Samples were inserted at the bottom of the 4 mm NMR rotor for semi-solid NMR analysis with forceps and push down with the help of pins. The rest of the rotor was filled with D_2_O and kept at 4°C until the solid NMR analysis took place. Liver samples collected for histological visualization were stained with a standard haematoxylin and eosin protocol.
