# Supplementary material for: NMR metabolomics identifies over 60 biomarkers associated with Type II Diabetes impairment in db/db mice
Source: Metabolomics. 2019 Jun 10;15(6):89. doi: 10.1007/s11306-019-1548-8 (PMC6556514; doi:10.1007/s11306-019-1548-8)
Supplement: Supplementary file 2 — Supplementary material 2 (DOCX 234 kb) [file 11306_2019_1548_MOESM2_ESM.docx]

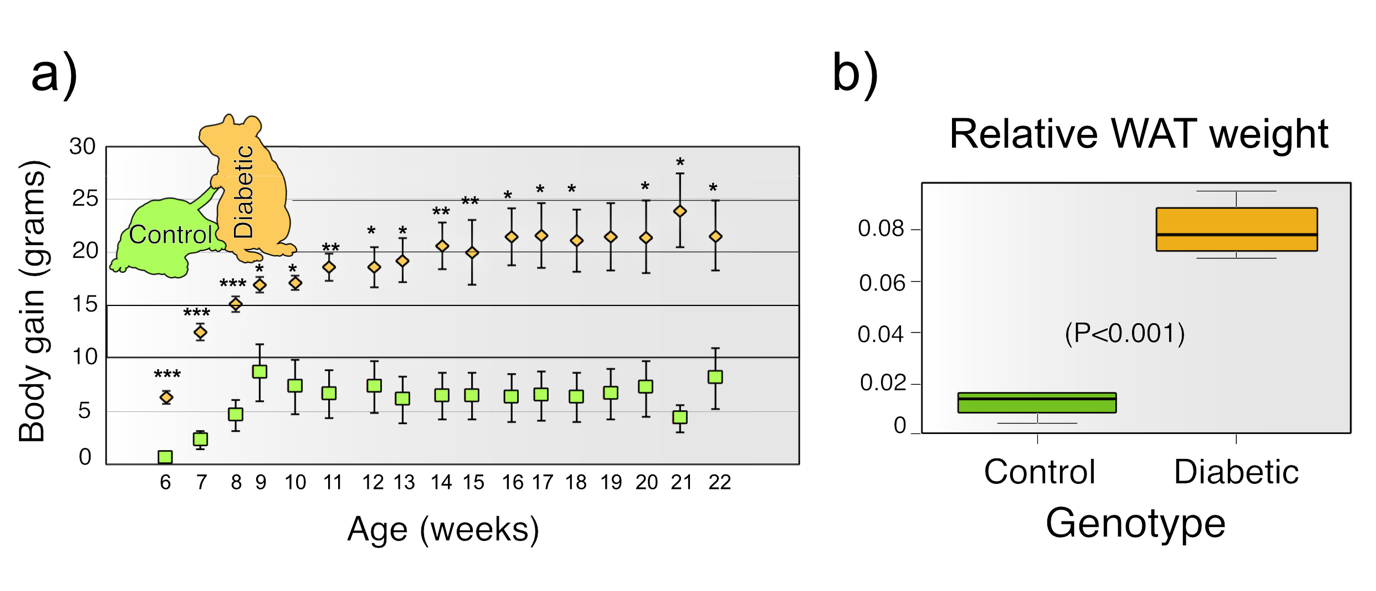


**S2_Fig 1:** Body weight gain in diabetic and control groups. a: A One-way ANOVA (n=12) showed that differences between body weight gain (W) of diabetic individuals (orange) and control ones (green) was statistically significant (*p*<0.01). b: Relative WAT weight was statistically higher in diabetic individuals than in controls (*p*<0.001).
