# Supplementary material for: NMR metabolomics identifies over 60 biomarkers associated with Type II Diabetes impairment in db/db mice
Source: Metabolomics. 2019 Jun 10;15(6):89. doi: 10.1007/s11306-019-1548-8 (PMC6556514; doi:10.1007/s11306-019-1548-8)
Supplement: Supplementary file 3 — Supplementary material 3 (DOCX 62161 kb) [file 11306_2019_1548_MOESM3_ESM.docx]

Small intestine was divided in three anatomical sections, duodenum, jejunum and ileum. The **duodenum** from diabetic individuals was characterized by increased levels of alanine, glutamate, glycine, isoleucine, lactate, leucine, taurine, threonine and valine (S2_Fig1, panels a and b; R^2^Y= 0.72, Q^2^Y=0.53; n=10). The **jejunum** from diabetic individuals was characterized by increased levels leucine, lipids and lysine, while choline, creatine glycolate and taurine were decreased (S2_Fig1, panels c and d; R^2^Y= 0.75, Q^2^Y=0.64, n=10). The diabetic **ileum** showed higher levels of lipids, and lower levels of alanine, arginine, aspartate, creatine, glutamate, taurine and uracil (S2_Fig1, panels e, f and g; R^2^Y= 0.83, Q^2^Y= 0.44; n=12).

**S3_Fig 1:** **Duodenum**: Plot of the scores against the cross validated scores generated from the O-PLS DA model calculated using all spectra as a matrix (n=10) of independent variables and genetic background as predictors (R^2^Y= 0.72, Q^2^Y=0.53). **Jejunum**: Plot of the scores against the cross validated scores generated from the O-PLS DA model calculated using all spectra as a matrix (n=10) of independent variables and genetic background as predictors (R^2^Y= 0.74, Q^2^Y=0.62). **Ileum**: Plot of the scores against the cross validated scores generated from the O-PLS DA model calculated using all spectra as a matrix (n=12) of independent variables and genetic background as predictors (R^2^Y= 0.83, Q^2^Y= 0.44).

Large intestine was divided in three sections, proximal, transversal and distal colon. The **proximal colon** from diabetic individuals was characterized by higher levels of lipids, while choline, creatine and taurine were reduced (S2_Fig2, panels a and b; R^2^Y= 0.77, Q^2^Y=0.44; n=11). In transversal colon, diabetic individuals were metabolically characterized by higher levels of butyrate and lower levels of creatine, leucine, O-phosphoethanolamine, phenylalanine, tyrosine and valine (S2_Fig2, panels c and d; R^2^Y= 0.77, Q^2^Y=0.40; n=10). Finally, in **distal colon**, diabetic individuals had higher levels of acetate, butyrate, glucose and isobutyrate; and lower levels of alanine, aspartate, creatine, glutamate, glycine, histidine, phenylalanine, tyrosine and uracil (S2_Fig2, panels d, f and g; R^2^Y= 0.78, Q^2^Y=0.43; n=11).

**S3_Fig2: Proximal colon:** Plot of the scores against the cross validated scores generated from the O-PLS DA model calculated using all spectra as a matrix (n=11) of independent variables and genetic background as predictors (R^2^Y= 0.77, Q^2^Y=0.44). **Transversal colon:** Plot of the scores against the cross validated scores generated from the O-PLS DA model calculated using all spectra as a matrix (n=10) of independent variables and genetic background as predictors (R^2^Y= 0.77, Q^2^Y=0.40). **Distal colon:** Plot of the scores against the cross validated scores generated from the O-PLS DA model calculated using all spectra as a matrix (n=11) of independent variables and genetic background as predictors (R^2^Y= 0.78, Q^2^Y=0.43).
