# Supplementary material for: NMR metabolomics identifies over 60 biomarkers associated with Type II Diabetes impairment in db/db mice
Source: Metabolomics. 2019 Jun 10;15(6):89. doi: 10.1007/s11306-019-1548-8 (PMC6556514; doi:10.1007/s11306-019-1548-8)
Supplement: Supplementary file 4 — Supplementary material 4 (DOCX 13453 kb) [file 11306_2019_1548_MOESM4_ESM.docx]

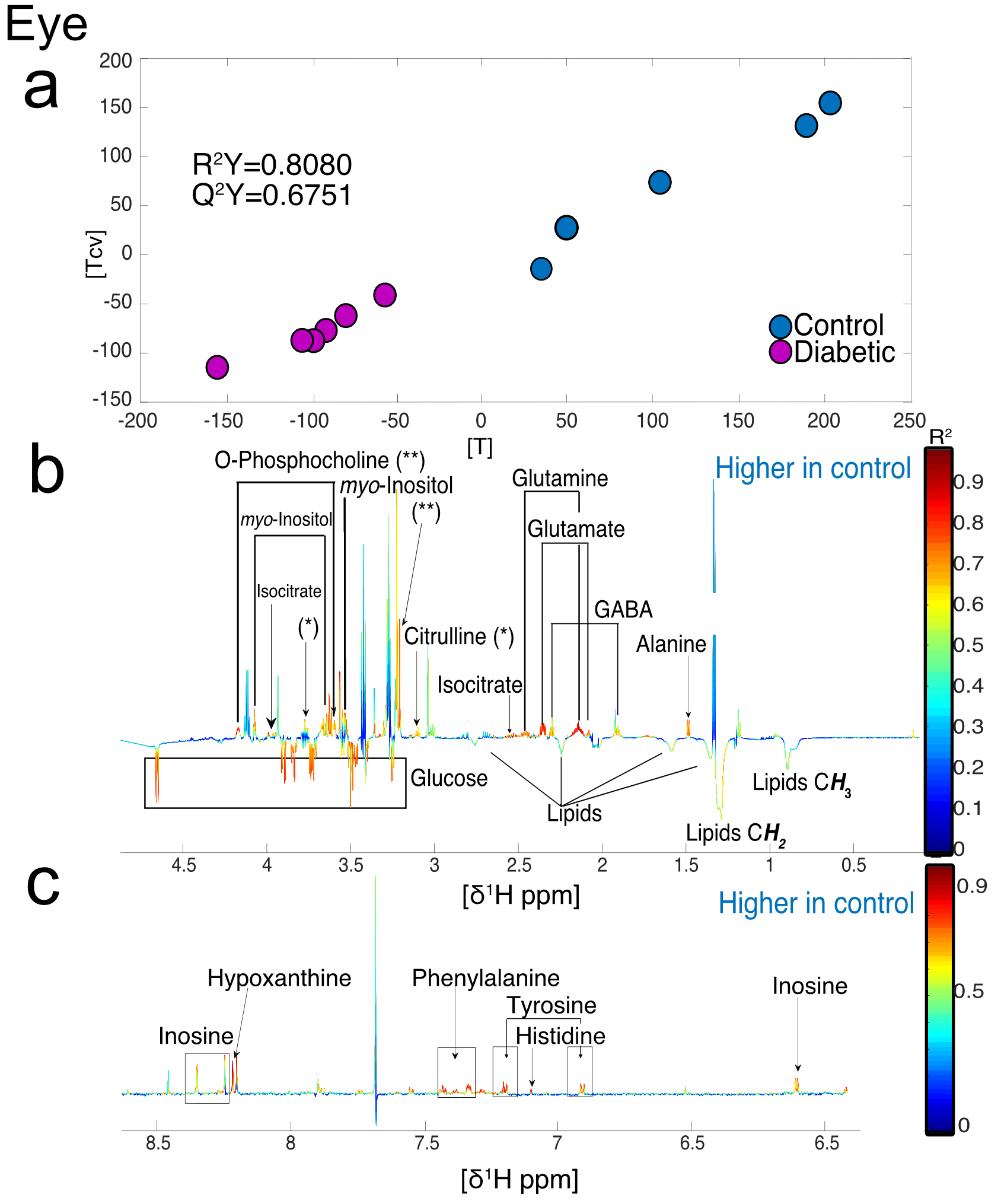


**S4_Fig 1:** Plot of the scores against the cross validated scores generated from the O-PLS DA model calculated using all eye spectra as a matrix (n=11) of independent variables and genetic background as predictors (R^2^Y= 0.81, Q^2^Y= 0.67).
