# Supplementary material for: NMR metabolomics identifies over 60 biomarkers associated with Type II Diabetes impairment in db/db mice
Source: Metabolomics. 2019 Jun 10;15(6):89. doi: 10.1007/s11306-019-1548-8 (PMC6556514; doi:10.1007/s11306-019-1548-8)
Supplement: Supplementary file 6 — Supplementary material 6 (DOCX 250 kb) [file 11306_2019_1548_MOESM6_ESM.docx]

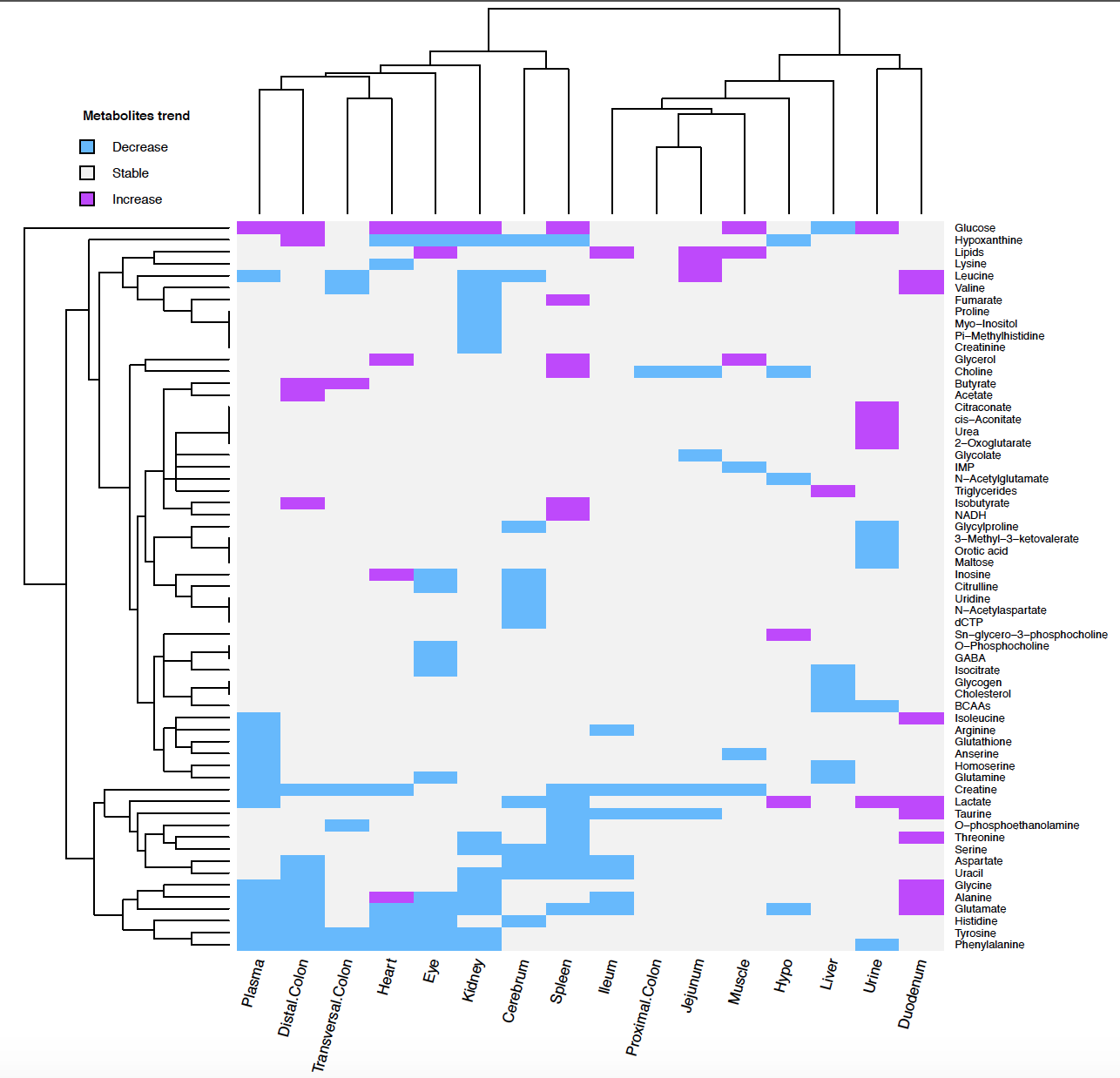


**S6_Fig 1:** Heat-map showing clusters of matrices and metabolites. Purple colour indicates an increase of the metabolite and blue colour a decrease of the metabolite in diabetic individuals compared to control ones.
